# Peptides-Derived from Thai Rice Bran Improves Endothelial Function in 2K-1C Renovascular Hypertensive Rats

**DOI:** 10.3390/nu7075252

**Published:** 2015-07-15

**Authors:** Orachorn Boonla, Upa Kukongviriyapan, Poungrat Pakdeechote, Veerapol Kukongviriyapan, Patchareewan Pannangpetch, Supawan Thawornchinsombut

**Affiliations:** 1 Department of Physiology, Faculty of Medicine, Khon Kaen University, Khon Kaen 40002, Thailand; E-Mails: kukkaiorachorn@gmail.com (O.B.); ppoung@kku.ac.th (P.P.); 2 Department of Pharmacology, Faculty of Medicine, Khon Kaen University, Khon Kaen 40002, Thailand; E-Mails: veerapol@kku.ac.th (V.K.); patc_pan@kku.ac.th (P.P.); 3 Department of Food Technology, Faculty of Technology, Khon Kaen University, Khon Kaen 40002, Thailand; E-Mail: suptha@kku.ac.th

**Keywords:** 2K-1C renovascular hypertension, angiotensin converting enzyme, endothelial function, nitric oxide, oxidative stress, rice bran peptides, Thai rice bran, vasorelaxation

## Abstract

In recent years, a number of studies have investigated complementary medical approaches to the treatment of hypertension using dietary supplements. Rice bran protein hydrolysates extracted from rice is a rich source of bioactive peptides. The present study aimed to investigate the vasorelaxation and antihypertensive effects of peptides-derived from rice bran protein hydrolysates (RBP) in a rat model of two kidney-one clip (2K-1C) renovascular hypertension. 2K-1C hypertension was induced in male Sprague-Dawley rats by placing a silver clip around the left renal artery, whereas sham-operated rats were served as controls. 2K-1C and sham-operated rats were intragastrically administered with RBP (50 mg·kg^−1^ or 100 mg·kg^−1^) or distilled water continuously for six weeks. We observed that RBP augmented endothelium-dependent vasorelaxation in all animals. Administration of RBP to 2K-1C rats significantly reduced blood pressure and decreased peripheral vascular resistance compared to the sham operated controls (*p <* 0.05). Restoration of normal endothelial function and blood pressure was associated with reduced plasma angiotensin converting enzyme (ACE), decreased superoxide formation, reduced plasma malondialdehyde and increased plasma nitrate/nitrite (*p <* 0.05). Up-regulation of eNOS protein and down-regulation of p47^phox^ protein were found in 2K-1C hypertensive rats-treated with RBP. Our results suggest that RBP possesses antihypertensive properties which are mainly due to the inhibition of ACE, and its vasodilatory and antioxidant activity.

## 1. Introduction

Hypertension is a common risk factor of cardiovascular disease. The incidence of hypertension is rising rapidly, and is increasing in Thailand and throughout the world [1]. The increase in cardiovascular risk inherent to hypertension leads to premature morbidity and mortality. It has been demonstrated that hypertension has a strong association with the formation of reactive oxygen species (ROS) [2]. ROS which are mainly produced by vascular cells are implicated in various cardiovascular diseases including hypertension. Importantly, inactivation of nitric oxide (NO) by superoxide (O_2_^•−^) is a key factor in reducing NO bioavailability and the consequent development of endothelial dysfunction in cardiovascular disease [3]. NO and angiotensin II (Ang II) have been reported to be key factors in regulation of blood pressure [4]. Inhibition of NO production stimulates the activation of the renin-angiotensin aldosterone system (RAAS) [4]. The RAAS plays an important role in the pathology of hypertension, cardiovascular health in general, and renal function [5]. Therefore, the balance between Angiotensin II (Ang II) and NO is an important element in the prevention and/or treatment of hypertension.

It is generally known that diet and lifestyle modification are among the most effective tools to prevent hypertension and maintain normal blood pressure. In this respect, the search for diet-related prevention of hypertension is obviously of interest within the scope of functional foods. Among these, food protein-derived angiotensin-converting enzyme (ACE) inhibitory peptides have received much attention for prevention of hypertension as well as for therapeutic purposes [6,7,8]. Although the ACE inhibitory potencies of these peptides are not as great as the ACE inhibitor drugs commonly used for treatment of hypertension, they are naturally derived from food protein sources. And are considered to be milder and safer without the side effects associated with the drugs.

Antihypertensive peptides are the most extensively studied of all the food protein, highlighting their importance in human health and disease prevention and treatment [9,10]. Among the antihypertensive peptides, peptides-derived from rice bran protein have received particular attention. Rice (*Oryza Sativa* L.) is the second largest cereal crop produced worldwide and the major dietary staple for more than half of the world’s population, mostly in Asian countries. Rice bran is a major by-product of the rice milling process. Rice bran contains a number of nutrients and bioactive compounds, including proteins, dietary fiber and phytochemicals with potent antioxidant, anti-diabetic, anti-dyslipidemic and anti-inflammatory activities, which have been demonstrated in both *in vitro* and *in vivo* experiments [11,12,13,14,15]. Rice bran protein is an important constituent accounting for 10%–15% by weight of rice bran and consists of 37% water-soluble, 31% salt-soluble, 2% alcohol-soluble, and 27% alkali-soluble storage proteins [16]. In recent years, the potential peptides produced by enzymatic hydrolysis of cereal proteins have received considerable attention due to their health-benefitting bioactivities. It has been demonstrated that peptides-derived from rice bran protein hydrolysates (RBP) possess free radical scavenging and anti-oxidative activities [17]. Moreover, RBP has also been shown to inhibit ACE activity *in vitro* and cause a reduction in systolic blood pressure in spontaneously hypertensive rats after a single oral administration [7]. However, the blood pressure lowering effect of RBP in long-term treatment has not yet been reported.

Based on previous reports of the *in vitro* ACE inhibitory activity of RBP, the present study has used two-kidney, one-clip (2K-1C) renovascular hypertension in the rat as an experimental model to investigate the antihypertensive effect of RBP. The development of hypertension in this model is mainly due to a high production of Ang II, which is generated through the activation of ACE by converting the inactive decapeptide Ang I to the potent vasoconstriction octapeptide Ang II [18]. Given the central role of ACE in activity of RBP, the present study, therefore, investigated the antihypertensive and vasorelaxation effects of RBP in 2K-1C renovascular hypertensive rats.

## 2. Experimental Section

### 2.1. Preparation of Rice Bran Protein Hydrolysates

Rice bran from jasmine rice (Hom Mali 105), defatted with cold-press extraction, was supplied from the Community Organic Produces Enterprise, Lopburi, Thailand. RBP were prepared according to a previously described method [19]. Briefly, rice bran was suspended in distilled water, adjusted to pH 11.0 and centrifuged at 5000× *g* for 30 min. The supernatant was adjusted to pH 4.5 for precipitation. The precipitate was suspended in distilled water at pH 7.0 and proteolysis was carried out using a commercial enzyme, Protease G6 (Genencor International Inc., Palo Alto, CA, USA) at pH 8.0, 55 °C for 4 h. Thereafter, the enzyme was inactivated by heating to 85 °C for 15 min. After centrifugation, the protein hydrolysates were freeze-dried. The yield of crude RBP powder was 8.8% by weight from the defatted rice bran. The crude RBP was suspended in distilled water and subjected to ultrafiltration using a membrane with molecular weight cut-off 50 kDa. The filtrate containing the low molecular weight RBP peptides was freeze-dried and stored in air-tight containers kept at −20 °C. The protein content, crude fat, moisture and total phenolic content were analyzed according the established methods [20], Association of Official Agricultural Chemists (AOAC) [21]. The composition of RBP was; protein content (in RBP low molecular weight): 46.6 g/100 g powder, crude fat: 16.32%, moisture: 2.01% and total phenolic content: 39.1 mg of Gallic acid per g extract.

### 2.2. Animals

Male Sprague-Dawley rats (160–180 g), obtained from the National Laboratory Animal Center, Mahidol University, Salaya, Nakornpathom, were used in this study. Animals were housed at the Northeast Laboratory Animal Center (Khon Kaen University, Khon Kaen, Thailand). They were maintained in a temperature-controlled room (25 ± 2 °C) with a 12-h dark/light cycle for a week before starting the experiments. The experimental protocols were reviewed and approved by the Institutional Animal Ethics Committee, Khon Kaen University, Thailand.

### 2.3. Induction of 2K-1C Renovascular Hypertension

Induction of 2K-1C hypertension was carried out according to the method originally described by Goldblatt *et al.* [22]. Briefly, rats were anesthetized with an intraperitoneal injection of pentobarbital sodium (50 mg·kg^−1^), a retroperitoneal flank incision was made and the left renal artery was exposed and cleared. A U-shaped silver clip (0.2 mm. i.d.) was placed around the renal artery and secured in place. The contralateral kidney was left intact. The incision was sutured and the animals were allowed to recover from anesthesia and returned to their cages. Sham-operated animals underwent the same surgical procedure without the clip placement. All surgery was performed under aseptic conditions. Animals with systolic blood pressure (SBP) greater than 140 mmHg measured by the tail-cuff method (see below) one week after clip placement were defined as hypertensive.

### 2.4. The Experimental Protocol

After recovery from surgery for one week, sham operated animals were randomly assigned to one of two control groups (sixteen animals in each): sham + deionized water (DI), sham + RBP 100 mg·kg^−1^. Hypertensive animals were assigned to one of three groups: 2K-1C + DI, 2K-1C + RBP 50 mg·kg^−1^, and 2K-1C + 100 mg·kg^−1^, respectively. Rats were intragastrically administered with RBP (50 mg·kg^−1^ or 100 mg·kg^−1^) or DI (1.5 mL·kg^−1^), as vehicle, for six weeks. Eight animals in each group were used for *in vitro* assessment of vascular reactivity and the remaining eight were used for *in vivo* hemodynamic measurements and biochemical evaluations. The doses of RBP used in this study were based on our previous study which showed that they were sufficient to reduce blood pressure in l-NAME hypertensive rats [23]. The SBP was measured on the day before renal artery clipping (regarded as baseline data), one week after surgery and weekly during treatment using the tail-cuff phletysmography (Blood pressure analyzer, model 179; IITC Life Science Inc., Woodland Hills, CA, USA).

### 2.5. In Vitro Assessment for the Effect of RBP on Vascular Reactivity

Six weeks after treatment, rats were sacrificed by an overdose of pentobarbital sodium. Aortic rings were set up in organ baths, and mechanical activity was measured as previously described [24]. Endothelium-dependent and endothelium-independent vasodilatation was studied by measuring the vasorelaxation-induced by acetylcholine (ACh) and sodium nitroprusside (SNP) at doses ranging from 10^−9^ to 10^−5^ M in the aortic rings pre-contracted with phenylephrine (1 μM).

To assess vascular reactivity in the mesenteric artery beds, the main branch of the superior mesenteric artery was rapidly excised after death. The vessel was identified, cleaned of connective tissue and cannulated with a blunted hypodermic needle. The mesenteric artery bed was placed on a stainless steel grid (7 cm × 5 cm) in a warm humid chamber (37 °C) and perfused at a constant flow rate of 5 mL·min^−1^, using a peristaltic pump (Cole-Palmer Instruments, Vernon Hills, IL, USA) with Krebs solution composed of the following (mM): NaCl 118.2, KCl 4.7, KH_2_PO_4_ 1.2, MgSO_4_·7H_2_O 1.18, Glucose 11.0, NaHCO_3_ 25 and CaCl_2_·2H_2_O 1.25 (pH 7.4). The solution was maintained at 37 °C and continually gassed with 95% O_2_ and 5% CO_2_. Mean perfusion pressure was monitored using a pressure transducer and the data recorded using a BIOPAC system (BIOPAC Systems Inc., Goleta, CA, USA).

After a 30 min equilibration period, methoxamine was added to raise tone in each preparation (50–70 mmHg above baseline) before endothelial function testing, by using 1 μM ACh. Under methoxamine raised tone conditions, ACh and SNP at doses ranging from 10^−9^ M to 10^−4^ M were directly injected into the proximal tube connected to the arterial cannula with an infusion pump. A volume of 100 μL for each concentration was injected at the rate of 10 μL per second. The concentration-response curves to ACh and SNP obtained from the aortic rings and mesenteric artery beds preparations were obtained.

### 2.6. In Vivo Assessment for the Effect of RBP on Hemodynamics and Biochemical Parameters

#### 2.6.1. Measurement of Hemodynamic Status

After six weeks of treatment, rats were anesthetized with pentobarbital sodium (60 mg·kg^−1^; i.p.) and a tracheotomy was performed to allow spontaneous breathing. The left femoral artery was exposed and cannulated and the cannula was connected to a pressure transducer for monitoring blood pressure (BP) and heart rate (HR), using the AcqKnowledge Data Acquisition System, (BIOPAC Systems Inc., Goleta, CA, USA). Hindlimb blood flow (HBF) was continuously measured by placing a cuff type electromagnetic flow probe (4 mm internal circumference) around the abdominal aorta, which was connected to an electromagnetic flowmeter (Carolina Medical Electronics Inc., East Bend, NC, USA). Hindlimb vascular resistance (HVR) was calculated from the mean arterial pressure (MAP) divided by HBF. Blood samples were withdrawn from the abdominal aorta for assays of ACE level and oxidative stress makers. Thereafter, the carotid arteries and aortas were rapidly excised and used for measurement of O_2_^•−^ production and Western blot analysis of eNOS and p47 ^phox^ NADPH oxidase subunit.

#### 2.6.2. Assay of Vascular O_2_^•−^ Production

O_2_^•−^ production in the carotid artery was measured using a lucigenin-enhanced chemiluminescence method as previously described [25]. In brief, the isolated carotid arteries were immediately placed in ice-cold Krebs solution, cut into rings 4 mm in length and incubated in a sample tube with Krebs salt solution at 37 °C for 30 min. Then the lucigenin was added into the tube, and placed in luminometer (Turner Biosystems Inc., Sunnyvale, CA, USA). The photon counts were integrated every 15 s for 5 min and averaged. The vessels were dried at 45 °C for 24 h, for determination of dry weight. O_2_^•−^ production in vascular tissues was expressed as relative light unit counts per minute per milligram of dry tissue weight.

#### 2.6.3. Assays of Nitrate/Nitrite, Malondialdehyde and Protein Carbonyl

Accumulation of nitrate and nitrite, the breakdown products of NO, was measured in plasma samples using a previously described method [25]. Again, following a previously described method [26], plasma malondialdehyde (MDA) was determined by measuring thiobarbituric acid reactive substances, and plasma protein carbonyl was assessed by measuring the formation of carbonyl groups after reaction with 2,4-dinitrophenylhydrazine.

#### 2.6.4. Assay of ACE Activity

Plasma ACE activity was determined using the *o*-phthalaldehyde (OPA)-chromogenic reaction for histidyl-leucine following previously described methods [24,27].

#### 2.6.5. Western Blot Analysis

Western blotting was performed on the aortic homogenates as previously described [25,28]. In brief, the thoracic aortas were homogenized in cell lysis buffer (Cell Signaling Technology Inc., Danvers, MA, USA) and centrifuged at 4 °C and 12,000 rpm for 30 min. The supernatant was collected and the protein content was analyzed by the Bradford dye-binding method [29]. Protein (30 μg) per sample was resolved by electrophoresis on 10% sodium dodecyl sulfate polyacrylamide gel and transferred onto a polyvinylidene difluoride membrane. Immunoblotting was performed using a specific primary antibody of either mouse monoclonal anti-endothelial NOS (eNOS) (1:2000 dilution; BD Biosciences, San Jose, CA, USA) or mouse monoclonal anti-p47^phox^ (1:1500 dilution; Santa Cruz Biotechnology, Indian Gulch, CA, USA). The membranes were washed and incubated for 2 h at room temperature with the secondary antibody horseradish peroxidase goat anti-mouse IgG (1:2000 dilution; Santa Cruz Biotechnology). Bands were visualized by enhanced chemiluminescence assay (Thermo Fisher Scientific Inc., Chicago, IL, USA). Specific eNOS or p47^phox^ NADPH oxidase and β-actin bands were imaged and captured using a digital imaging system for quantitative imaging of gels and blots (Imagequant 400, GE Healthcare, Pittsburgh, PA, USA). The intensity of the bands was normalized to β-actin expression from the same sample. The intensities were expressed as percentages of those from the aorta of normal controls.

### 2.7. Statistical Analysis

Results were expressed as mean ± SEM of measurements. The differences among various groups were compared by using one-way analysis of variance (ANOVA) followed by a post-hoc test with Student Newman-Keul’s test to determine the differences between groups. A *p* value of <0.05 was considered statistically significant.

## 3. Results

### 3.1. Effect of RBP on Vascular Reactivity

**Figure 1:**
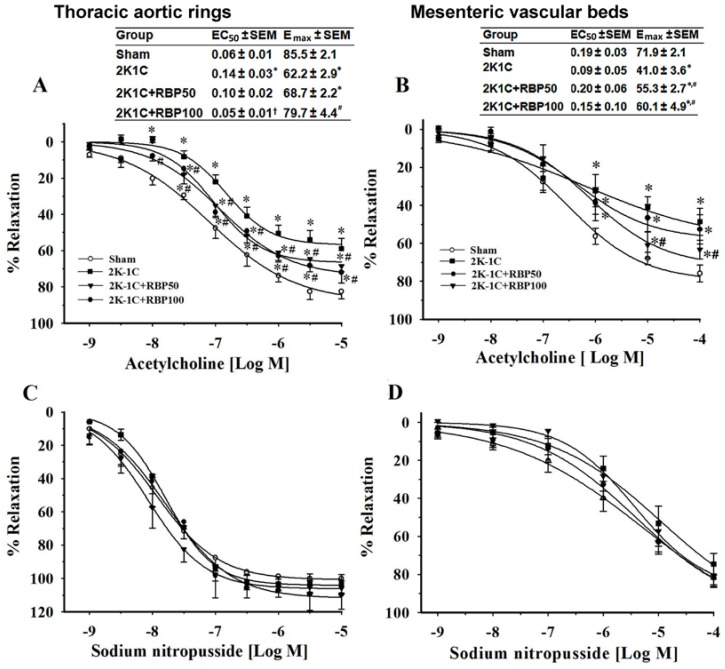
Effect of RBP on endothelium-dependent vasorelaxation induced by acetylcholine and endothelium-independent vasorelaxation induced by sodium nitroprussside in aortic rings pre-contracted with phenylephrine (**A**,**C**) and mesenteric artery beds pre-contracted with methoxamine (**B**,**D**). Data are mean values ± SEM; (*n =* 8/group), * *p* < 0.05 *vs.* sham-operated group, ^#^ *p* < 0.05 *vs.* 2K-1C group; Rice bran peptides, RBP; Maximal response, E_max_; half maximal effective concentration, EC_50_.

To evaluate endothelial function, endothelium-dependent vasorelaxation to ACh was examined both in the aortas and mesenteric arteries from the experimental groups. ACh-induced concentration-dependent relaxation was significantly reduced in aortic rings and mesenteric artery beds obtained from 2K-1C hypertensive rats compared to sham controls (*p <* 0.05; Figure 1A and B). In the thoracic aortas of 2K-1C rats, both maximal response (intrinsic efficacy) and potency (strength) of the vascular response to ACh were significantly decreased when compared to sham-operated controls (E_max_, 62% *vs.* 86%; EC_50_, 0.14 μM *vs.* 0.06 μM, respectively). In comparison with the sham-operated controls, the maximal response to ACh of the mesenteric artery of 2K1C rats was significantly decreased, (E_max_ 41% *vs.* 72%), whereas the EC_50_ was unchanged (0.09 μM *vs.* 0.19 μM). Meanwhile, endothelium-independent vasorelaxation induced by the NO donor, SNP did not differ between experimental groups (Figure 1C and D). The results suggest that the 2K-1C model affects endothelial function by altering the ability of blood vessels to respond to ACh and the effect is stronger in the thoracic aorta. However, changes in the endothelial cells in the two types of vessel at the molecular level were not explored in this study. Interestingly, RBP significantly restored the impairment in endothelial-dependent vasorelaxation to ACh in both the aortas and mesenteric arteries of 2K-1C rats (*p* < 0.05; Figure 1A and B). This restoration was particularly evident in rats treated with the high dose of RBP (100 mg·kg^−1^), where the E_max_ and EC_50_ came back to the control values.

### 3.2. Effect of RBP on Hemodynamic Status

**Figure 2:**
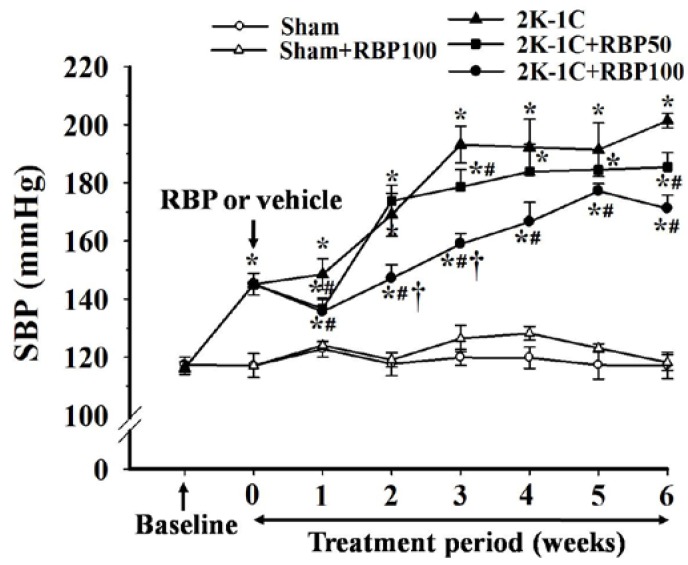
Effect of RBP on systolic blood pressure measured by tail-cuff plethysmography before and after renal artery clipping. Rice bran peptides, RBP; Systolic blood pressure, SBP. Data are mean ± SEM; (*n =* 8/group), * *p* < 0.05 *vs.* sham-operated group, ^#^ *p* < 0.05 *vs.* 2K-1C group and **^†^** *p <* 0.05 *vs.* 2K-1C + RBP50 group.

**Figure 3:**
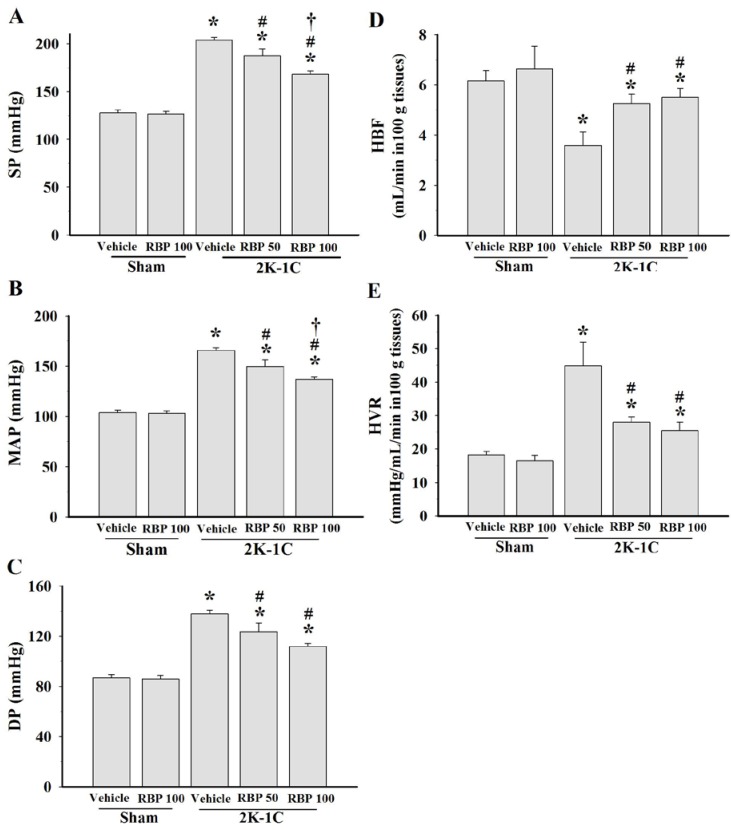
Effect of RBP on systolic blood pressure (**A**); mean arterial pressure (**B**); diastolic blood pressure (**C**); hindlimb blood flow (**D**) and hindlimb vascular resistance (**E**). Data are mean values ± SEM; (*n =* 8/group), * *p <* 0.05 *vs.* sham-operated group, ^#^ *p <* 0.05 *vs.* 2K-1C group and **^†^** *p <* 0.05 *vs.* 2K-1C + RBP50 group; Rice bran peptides, RBP.

Before clipping the renal artery, there were no significant differences of baseline SBP in all experimental groups (Figure 2). SBP of 2K-1C hypertensive rats was significant elevated after renal artery clipping throughout the experimental period. Oral administration of RBP significantly reduced SBP of 2K-1C rats compared to untreated 2K-1C rats (*p* < 0.05; Figure 2). Hemodynamic measurements made on anaesthetized animals 6 weeks after the start of treatment with RBP at both doses significantly attenuated the changes of systolic pressure, diastolic pressure and mean arterial pressure in the clipped rats (*p* < 0.05, Figure 3A–C). Hindlimb blood flow was decreased, whereas the calculated hindlimb vascular resistance was increased in 2K-1C hypertensive rats compared with sham-operated controls (*p* < 0.05, Figure 3D,E). The hemodynamic disturbance was reversed by treatment with 50 and 100 mg·kg^−1^ RBP for six weeks (Figure 3D,E). Although all hemodynamic parameters still differed from those of the control animals, these results suggest that RBP exhibits antihypertensive effects.

### 3.3. Effect of RBP on NO Production

**Figure 4:**
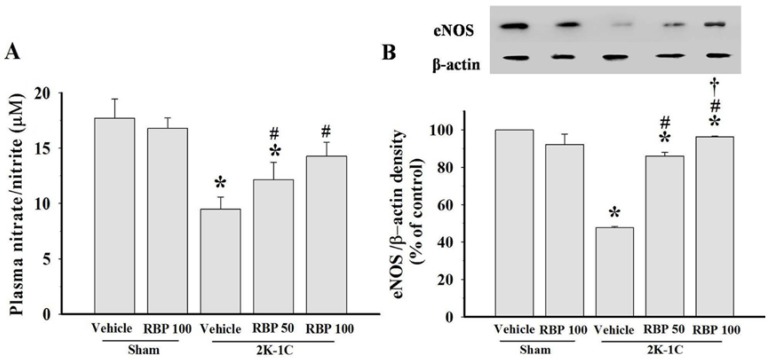
Plasma nitrate/nitrite levels (**A**) and eNOS protein expression and densitometric analysis in thoracic aortas (**B**) in all experimental groups. Data are mean values ± SEM. (*n =* 8/group), * *p <* 0.05 *vs.* sham-operated group, ^#^ *p <* 0.05 *vs.* 2K-1C group and **^†^** *p <* 0.05 *vs.* 2K-1C + RBP50 group. Rice bran peptides, RBP.

Impairment of endothelial vasodilation in 2K-1C hypertension is confirmed by a reduction of plasma nitrate/nitrite concentration, and a down-regulation of eNOS protein expression in the aortas of 2K-1C rats (*p* < 0.05, Figure 4A and B). RBP significantly improved endothelial dysfunction of 2K-1C rats by increasing nitrate/nitrite levels and up-regulation of eNOS expression (*p* < 0.05, Figure 4A and B).

### 3.4. Effect of RBP on O_2_^•−^ Production and Oxidative Stress

**Figure 5:**
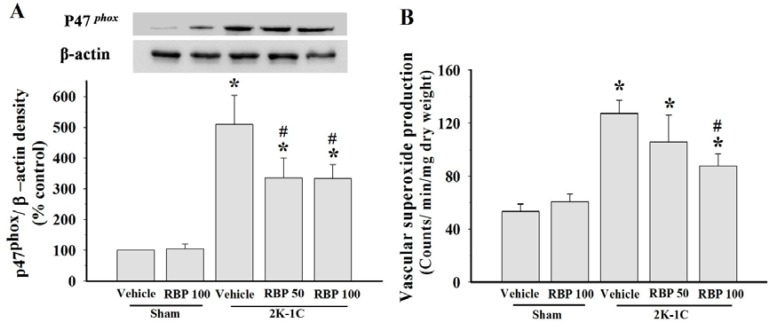
p47^phox^ protein expression and densitometric analysis in thoracic aortas (**A**) and vascular superoxide production (**B**) in all experimental groups. Data are mean values ± SEM; (*n =* 8/group), * *p <* 0.05 *vs.* sham-operated group, ^#^ *p <* 0.05 *vs.* 2K-1C group and **^†^** *p <* 0.05 *vs.* 2K-1C + RBP50 group; Rice bran peptides, RBP.

**Figure 6:**
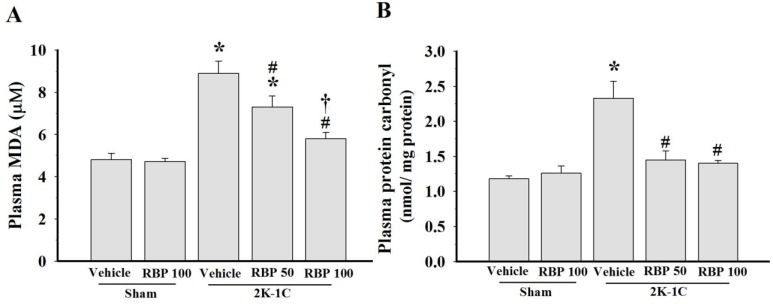
Effect of RBP on plasma malondialdehyde (**A**) and plasma protein carbonyls (**B**) concentrations. Data are mean values ± SEM; (*n =* 8/group), * *p <* 0.05 *vs.* sham-operated group, ^#^ *p <* 0.05 *vs.* 2K-1C group and **^†^** *p <* 0.05 *vs.* 2K-1C + RBP50 group; Rice bran peptides, RBP.

Increased vascular O_2_^•−^ production with up-regulation of p47^phox^ NADPH oxidase subunit in the aortas were found in 2K-1C rats when compared with sham-operated controls (*p < 0.05*, Figure 5A and B), indicating increased ROS production in this model of renovascular hypertension. Treatment with RBP significantly reduced p47^phox^ protein expression and O_2_^•−^ generation in the vascular tissues (*p* < 0.05, Figure 5A and B). Further confirming the association of increased oxidative stress with hypertension, we found that 2K-1C animals had higher levels of plasma MDA and protein carbonyl than the sham-operated controls (*p* < 0.05, Figure 6A and B). Treatment with RBP significantly decreased the MDA and protein carbonyl levels of 2K-1C rats (*p* < 0.05, Figure 6).

### 3.5. Effect of RBP on ACE Activity

**Figure 7:**
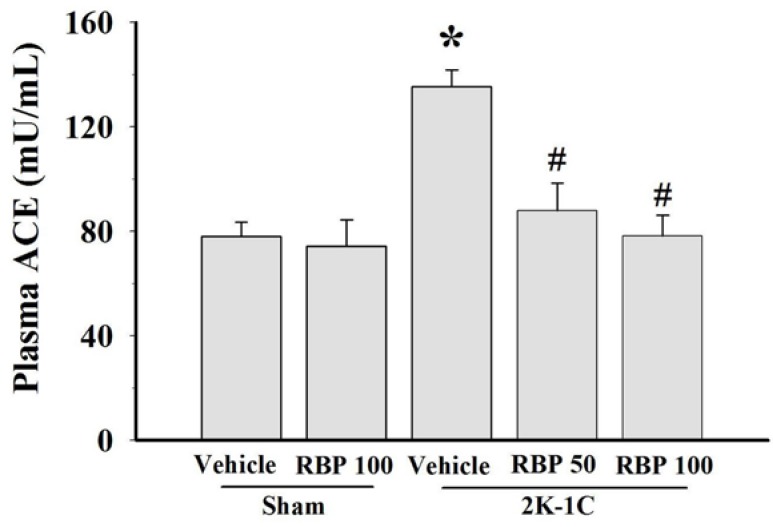
Effect of RBP on plasma angiotensin converting enzyme activity. Data are mean values ± SEM; (*n =* 8/group), * *p <* 0.05 *vs.* sham-operated group, ^#^ *p <* 0.05 *vs.* 2K-1C group; Rice bran peptides, RBP.

ACE activity was measured in the plasma of 2K-1C and sham-operated controls. The plasma ACE level of 2K-1C rats was significantly higher than that of control rats, and treatment with RBP significantly reduced their plasma ACE level to normal concentration (*p* < 0.05, Figure 7). However, there was no change in plasma ACE concentration of sham-operated controls treated with RPB (Figure 7).

## 4. Discussion

This study has demonstrated that long-term administration of RBP reduced blood pressure, improved hemodynamic function, attenuated endothelial dysfunction and decreased oxidative stress of 2K-1C renovascular hypertensive rats. Apart from the ACE inhibitory activity, other mechanisms of action of RBP in this study are an increase in NO bioavailability through the enhancement of the eNOS pathway and alleviation of ROS formation through the suppression the NADPH oxidase system pathway.

The 2K-1C renovascular hypertension model used in this study is analogous to the renal artery constriction occurring in humans. It has been suggested that the hemodynamic and structural alterations seen in the 2K-1C model are caused by a higher level of Ang II generation in the established stage of hypertension [30]. Therefore, inhibition of ACE activity can decrease Ang II production and finally decrease blood pressure. Results of this study showed that plasma ACE activity was increased in 2K-1C, and the ACE level was decreased after treatment with RBP, suggesting that ACE inhibitory activity of RBP may be one of the mechanisms for its antihypertensive effects.

Previous studies have shown that ACE inhibitory peptides derived from enzymatic hydrolysis in different food proteins possess antihypertensive effects both in animals and in humans [31,32]. It has been found that rice dreg protein hydrolysates evinced *in vitro* ACE inhibitory activity and exhibited an antihypertensive effect in SHR and obese rats [6,7,15,33]. Moreover, several food proteins have been reported to be an important source of bioactive peptides with anti-oxidative, immune-modulating and hypocholesterolemic properties [34].

A variety of different peptides and peptide fragments with ACE inhibitory capabilities have been identified within many different plant food proteins, including rice protein, milk protein, soybean protein, and cereal grains [10,35,36,37]. Normally, people consume white rice or milled rice from which the husk and bran have been completely removed. People may benefit from consuming the whole grain or partially polished rice (Hom-Mali Thai rice in this study), while the rice bran hydrolysates are a potential source of food supplement for health promotion. It has been suggested that the bioactive peptides reduce blood pressure by various different mechanisms, such as ACE inhibition, antioxidant properties, a vasodilatory effect, modulating sympathetic nervous system activity and modulating vascular remodeling [38]. A previous study has demonstrated that increased oxidative stress in the early phase (2 weeks) of 2K-1C hypertension probably activates the matrix metalloproteinases (MMPs) and later promotes increases in their production, thus accelerating the vascular remodeling process with time [39]. Since RBP possesses strong antioxidant activity, it thus might attenuate the increase in MMPs and decrease vascular alterations of the 2K-1C rats, resulting in reduction of the blood pressure. However, more work is needed to identify its exact role. Although, most food proteins are degraded during their transit through the small intestine, they may demonstrate bioactive peptides throughout the whole intestine and they can display bioactivity in the small and large bowel. Moreover, a previous study has demonstrated that ACE inhibitory peptides present in the RBP are relatively resistant to digestion by gastrointestinal enzymes, as ACE inhibitory activity of the hydrolysate was slightly decreased after treatment with digestive enzymes [7], suggesting that rice protein hydrolysates can be absorbed intact through the intestine.

The vascular endothelial cell synthesizes NO from l-arginine via eNOS. It is one of the most important regulators of vascular tone. Decreased eNOS expression, increased expression of NADPH oxidase and the subsequent increased formation of O_2_^•−^ in the vascular wall may lead to reduce NO bioavailability, thereby causing endothelial dysfunction [2]. Moreover, increased oxidative stress has been recognized as one of the causes of hypertension in 2K-1C renovascular hypertension, and this is involved with endothelial dysfunction [24,40]. Consistent with others working on the same model [41,42,43], we observed impaired endothelium-dependent vasorelaxation to ACh and found that RBP could restore the vascular reactivity to ACh in 2K-1C rats. Improvement of endothelial function associated with RBP administration may be due to the activation of NO release from endothelial cells as evidenced by up-regulation of eNOS protein expression in the aorta and the increase of plasma nitrate/nitrite level. A similar effect on decreased SBP, enhanced endothelial function and increased aortic eNOS expression was found in SHR rats treated with fermented milk enriched tripeptides and pure tripeptides [33,44].

Another possible mechanism of the antihypertensive effect of RBP may be its pronounced antioxidant activity. In the vascular wall, ROS are produced by several enzyme systems including NADPH oxidase, uncoupled endothelial nitric oxide synthase (eNOS), xanthine oxidase and the mitochondrial electron transport chain. NADPH oxidase subunits are major sources of O_2_^•−^ production from molecular oxygen using NADPH as the electron donor [45]. In this study, increased O_2_^•−^ production was present in 2K-1C rats and this was associated with increased aortic p47^phox^ expression. Increased O_2_^•−^ from NADPH oxidase and increased degradation of NO by reaction with O_2_^•−^, thereby reduces NO bioavailability [2]. Thus, eNOS becomes uncoupled in 2K-1C hypertension, particularly when there is increased oxidative stress. The uncoupled eNOS produces O_2_^•−^ rather than NO. The increase in eNOS protein and decrease in p47^phox^ protein after RBP treatment suggest that RBP acts as an antioxidant, decreasing ROS generation and increasing NO bioavailability, thereby enhancing endothelial function and reducing the blood pressure. The increase in plasma nitrate/nitrite level after RBP treatment due to the metabolism of NO may be another mechanism of reduced blood pressure. In a previous study in 2K1C aortas, sodium nitrite was shown to inhibit vascular NADPH oxidase activity [46]. Moreover, increased oxidative stress as evidenced by increase of plasma MDA and protein carbonyl was also observed in the 2K-1C rats. Taken together, increased superoxide radicals, increased oxidative stress and decreased NO bioactivity may lead to endothelial dysfunction and hemodynamic disturbance (*i.e.*, reduced blood flow, increased peripheral vascular resistance and increased blood pressure) in this hypertension model. The results of this study indicate that RBP possesses antioxidant properties as shown by decreased lipid peroxidation and protein oxidation, decreased O_2_^•−^ production with down-regulation of p47^phox^ protein expression in 2K-1C-treated rats. Interestingly, it has been found that reduction in oxidative stress is associated with the improvement of endothelial function and decreased arterial blood pressure. Another study in obese rats-treated with rice bran enzymatic extraction also reported that rice bran extract restored endothelial function by reducing vascular NADPH oxidase expression and increasing eNOS protein [15]. The bioavailability of peptides with ACE-inhibitory activity derived from ingested foods in rodents may be different from humans. Therefore, any antihypertensive effect seen in rodents has to be validated in humans.

## 5. Conclusions

This study has provided evidence of RBP’s antihypertensive activity. Plausible mechanisms of this antihypertensive effect include ACE inhibitory activity, restoration of endothelial function, improvement of hemodynamic status, and reduction of oxidative stress in renal hypertensive rats. It follows that the bioactive peptides-derived from rice bran protein may be used as functional food products or nutraceutical ingredients for prevention and treatment of hypertension in humans.
